# Exploring metacognitive function in patients with opioid use disorder: A pilot study

**DOI:** 10.1111/papt.70081

**Published:** 2026-06-01

**Authors:** John Hanson Högberg, Björn Philips, Jonas Stålheim, Kristina Berglund

**Affiliations:** ^1^ Department of Psychology University of Gothenburg Gothenburg Sweden; ^2^ Department of Psychology Stockholm University Stockholm Sweden; ^3^ Department of Social Work University of Gothenburg Gothenburg Sweden

**Keywords:** longitudinal, metacognition, opioid maintenance treatment, opioid use disorder

## Abstract

**Objectives:**

Despite recognised cognitive deficits related to substance use, as well as high mortality and treatment attrition rates, metacognition has never been studied in patients with opioid use disorder (OUD) undergoing opioid maintenance treatment (OMT).

**Design:**

This exploratory pilot study used a quantitative longitudinal design to assess metacognition in patients with opioid use disorder at treatment initiation and at a one‐year follow‐up and to explore its association with executive functioning.

**Methods:**

Forty‐eight adults initiating OMT were enrolled in the study. All participants completed the Indiana Psychiatric Illness Interview (IPII), and recorded transcripts were analysed with the metacognition assessment scale (MAS‐A). Additionally, measures of executive function, as well as sociodemographic, psychiatric comorbidity and substance use, were obtained within two weeks of OMT initiation. IPII and MAS‐A were repeated at a one‐year follow‐up.

**Results:**

Participants displayed widespread performance of metacognition in the four subscales *self‐reflectivity, understanding others' minds, decentration* and notably, low levels of *mastery. Self‐reflectivity and mastery* were significantly positively correlated with verbal fluency. Higher levels of *decentration* were associated with fewer self‐reported executive function difficulties. No significant changes in metacognition were detected at the one‐year follow‐up.

**Conclusions:**

This longitudinal pilot study provides the first evidence of metacognitive functioning in OMT patients. Metacognitive performance was characterised by low levels of *mastery*, limited correlation with executive function and no improvement observed over the one‐year follow‐up period. The preliminary findings are interpreted and discussed with caution, emphasising the relevance of metacognition for future research and clinical interventions in OUD populations.

## METACOGNITION IN OPIOID MAINTENANCE PATIENTS

Opioid Use Disorder (OUD) is a global public health issue, associated with significant suffering, functional impairments and premature mortality, often due to opioid overdose (Ramey & Regier, 2019; Strang et al., 2020). Given its widespread prevalence across the world, it has increasingly been described as an “epidemic”. This opioid epidemic is reaping over 100,000 deaths globally each year (UNODC, 2023).

Opioid maintenance treatment (OMT) is considered the gold standard for treating OUD (Scott et al., 2021) and is strictly regulated in most countries (Strang et al., 2020). It constitutes a structured, long‐term form of care aimed at reducing illicit opioid use, preventing overdoses and fostering stability in patients' lives. The treatment framework includes pharmacotherapy, methadone and buprenorphine. Methadone is a long‐acting medication that fully activates opioid receptors, thereby reducing withdrawal symptoms and cravings in individuals with opioid dependence. Buprenorphine is a medication that partially activates opioid receptors, alleviating withdrawal and cravings while carrying a lower risk of overdose than full agonists. OMT comprises regular medical monitoring alongside psychosocial and psychological support, which may include cognitive‐behavioural therapy, psychodynamic therapy, case management and assistance related to housing, finances and employment. OMT has shown efficacy for recovery in terms of abstinence from opioids, mortality risk and improved overall health of patients suffering from opioid addiction (Strang et al., 2020). OMT has been shown to be associated with improvements in psychiatric symptoms and sense of coherence, reductions in psychological vulnerability and increased employment, suggesting an overall improvement in functioning (Öhlin et al., 2015). Despite its efficacy, many patients experience relapses, poor adherence to treatment and a retention rate of less than 50% after six months, which is associated with an increased risk of mortality (Strang et al., 2020).

These challenges highlight the need to better understand underlying mechanisms that may hinder treatment success. One potential factor is the presence of cognitive impairments, including deficits in metacognition, the capacity to reflect upon and regulate one's own cognitive and emotional states (Lysaker et al., 2019). Impaired metacognition may compromise an individual's ability to accurately assess their need for treatment, engage meaningfully in therapeutic processes and sustain behavioural change, thereby contributing to poor addiction treatment outcomes (Ramey & Regier, 2019). Impaired metacognitive capacity could potentially hinder treatment of OUD, as abilities involved in decision making, planning and understanding one's own strengths and limitations during the course of treatment are likely to enhance the chances of sustained recovery (Ramey & Regier, 2019). Despite the apparent relevance of metacognition in treatment contexts, it has never been investigated in patients with OUD, nor has it been examined whether metacognitive capacities may improve over the course of OMT (Strang et al., 2020).

Metacognition refers to the essential capacity for understanding thoughts, feelings and behaviour of oneself and others and for using this knowledge to master psychological problems (Lysaker et al., 2019). The theory of metacognition, developed by John H. Flavell in the 1970s through research on children's cognitive development and learning strategies, originated in developmental psychology and education but has since been increasingly applied in clinical research, especially in studies of schizophrenia (Flavell, 1979; Moritz & Lysaker, 2018). One definition of metacognition is: The set of skills necessary for: (a) identifying mental states and ascribing them to oneself and others on the basis of facial expressions, somatic states, behaviour and actions; (b) reflecting on and reasoning about mental states; (c) using information about mental states to decide, solve problems or psychological and interpersonal conflicts and master subjective suffering (Lolley, 2012). There are several approaches to investigating metacognition. One of the most used methods for examining more general metacognitive capacities, based on how individuals narrate and describe their lives and how they deal with difficulties, is the use of the Metacognition Assessment Scale–Abbreviated (MAS‐A). Individuals are interviewed using a semi‐structured interview that includes questions about their life experiences and coping strategies, and the responses are subsequently evaluated and quantified according to the complexity and level of abstraction reflected in different aspects of metacognition (Lysaker et al., 2019). The MAS‐A encompasses several aspects of metacognition, including *self‐reflectivity*, which involves the ability to reflect on one's thoughts and feelings and perceive them as products of internal and external circumstances; *understanding others' minds* is the ability to reflect on the thoughts and feelings of others; *decentration* refers to the capacity to recognise that reality extends beyond one's own perspective and that other individuals possess motives and desires that are separate and autonomous from one's own; *mastery* is the ability to utilise self‐knowledge in coping with psychological challenges. The MAS‐A scale has primarily been used in schizophrenia research to demonstrate that metacognition is a core feature contributing to a range of clinical symptoms and functional impairments (Lysaker, Dimaggio, Carcione, et al., 2010; Kukla & Lysaker, 2020). It has also been used in research on personality disorders, where studies have shown that metacognition plays a substantial role in explaining the difficulties experienced by individuals with personality disorders (PD) (Semerari et al., 2003). Furthermore, the severity of PD has been shown to correlate with the degree of metacognitive dysfunction (Semerari et al., 2014). Given the high rates of comorbidity, Dimaggio et al. (2015) proposed the use of the concept of metacognition in both research and clinical treatment of co‐occurring PD and substance use disorders (SUD).

A parallel higher‐order function in the brain that shares certain similarities with metacognition is executive functioning. Executive functioning comprises a range of cognitive processes associated with goal‐directed behaviour, long‐term planning, abstract reasoning, evaluating advantages and disadvantages before acting and the ability to inhibit impulses, among other capacities (Lysaker et al., 2008; Ramey & Regier, 2019). Metacognition and executive functioning may best be understood as systems of semi‐independent abilities (Lysaker et al., 2008). As the definitions indicate, both constructs refer to higher‐order cognitive processes that enable individuals to monitor and regulate their thinking and behaviour. Because both involve activity in the prefrontal cortex, they may be neurologically related; however, research also suggests that they engage partly distinct neural regions (Fleming & Dolan, 2012; Lysaker et al., 2008; Ramey & Regier, 2019). In substance use disorders, several studies have reported impairments in both metacognitive and executive processes, as well as disruptions in prefrontal cortex functioning; however, the overall evidence remains limited, and the nature of the relationships between these processes is still not well understood (Fleming & Dolan, 2012; Lysaker et al., 2008; Ramey & Regier, 2019). In a previous study by Högberg et al. (2025) using the same sample, executive functions were examined at baseline and at a one‐year follow‐up. The results showed that the patient group was heterogeneous in their executive abilities but, overall, performed below the level of the general population. Improved function was indicated in terms of verbal fluency, whereas cognitive flexibility and problem solving remained stagnant according to the longitudinal results. (Högberg et al., 2025). Metacognition has displayed relationships with executive function, such as cognitive flexibility, verbal fluency, working memory and problem solving in a group of schizophrenia patients (Lysaker et al., 2008). The study by Lysaker et al. (2008) showed that *self‐reflectivity* was positively linked to mental flexibility and cognitive insight, while *understanding others' minds* was positively linked to inhibitory control. In summary, the results indicated a relationship between the different domains of metacognition and executive function. To our knowledge, it has not yet been investigated whether executive functions are related to metacognition in a group of patients with OUD.

Opioid use affects the prefrontal cortex and alters several aspects of function (Barahmand et al., 2016; Delis et al., 2001; Ramey & Regier, 2019; Strang et al., 2020). These alterations are involved in the development of withdrawal and tolerance, as well as the gradual shift from voluntary consumption to obsessive‐compulsive behaviour characterised by a recurring preoccupation and repetitive, excessive substance use (Strang et al., 2020; Volkow et al., 2016). As described previously, medication‐assisted treatment for OUD involves the use of opioid agonists, which can dampen brain function and potentially contribute to impairments in both metacognition and executive functioning (Loeber et al., 2012; Mintzer et al., 2004). At the same time, these medications have a crucial general stabilising effect on patients' lives and greatly reduce the risk of overdose (Strang et al., 2020). OMT patients may differ from other SUD populations, as opioid‐based treatment may contribute to sustained cognitive impairment. Treatment of OUD would benefit from studying the relationship between executive function and metacognition, as poly‐substance abuse and psychiatric comorbidity may affect both functions. Treatment interventions could address either executive function, metacognition or a joint factor, but they need to be based on available research evidence, which is lacking.

To date, no study has specifically investigated metacognition in individuals with opioid use disorder (OUD) undergoing opioid maintenance treatment (OMT). To our knowledge, two studies have, however, examined metacognition in samples with substance use disorders (SUD). In a study comparing patients with schizophrenia and patients with SUD, the latter performed better in all metacognitive functions except *mastery*, the ability to utilise self‐knowledge when coping with psychological challenges. This indicated a specific weakness in the SUD group (Wasmuth et al., 2015). The results suggested an effect of concurrent pathologies, such as polysubstance use, which warranted research on how comorbid pathologies are connected to metacognitive function (Wasmuth et al., 2015). Another study investigating metacognitive *mastery* in SUD patients reported that lower levels of *mastery* were associated with more pronounced personality disorder traits and higher levels of alexithymia, the inability to identify and express emotions (Lysaker et al., 2014).

To summarise, previous research has demonstrated metacognitive deficits in patients with substance use disorders (SUD), but no study has specifically examined metacognition in OMT. Moreover, no research has investigated whether metacognitive capacity changes during OMT or how it relates to executive functions in OMT. The relationship between metacognition and executive functions in OMT patients remains unexplored, yet it is important to investigate this relationship in order to advance knowledge within both research and routine clinical practice.

### Aim

This exploratory pilot study aims to investigate metacognition in OUD patients starting OMT as well as to explore the possible association between metacognition and executive functioning in this patient group. The research questions are:

1. What level of metacognition do OUD patients exhibit at the start of OMT and at a one‐year follow‐up of OMT treatment?
2. What is the relationship between metacognition and executive function at the start of OMT?

### Participants

Participants were 48 patients (13 women and 35 men) starting opioid maintenance treatment, diagnosed with F11.2 opioid dependence. Their mean age was 40.7 years (*SD* = 10.3; range 23–66 years). Baseline demographic variables are summarised in Table 1. Psychiatric symptoms and types of personality disorders for participants are presented in Table 2. The average number of psychiatric diagnoses was 3.3 (*SD* = 2). The most frequent diagnoses were Depression, ADHD and Panic disorder. The average number of personality disorders was 5.9 (*SD* = 2.4). The most frequent personality disorders are Borderline, Antisocial and Narcissistic. Current drug use at baseline and follow‐up is presented in Table 3. At baseline measurement, all participants were using opioids, whereas opioid use had decreased markedly at the follow‐up assessment, with only six out of 41 participants still using opioids. The use of benzodiazepines remained relatively stable, with approximately half of the participants reporting use at baseline and at follow‐up. Around one in five participants used stimulants at both baseline and follow‐up. Cannabis use was relatively uncommon, with about one in ten participants reporting use at both time points. The average number of drugs used at admission was 4.1 (*SD* = 2.3) and had significantly been reduced at follow‐up to 1.9 (*SD* = 1.9) *t*(40) = 5.31, *p* < .001. The effect size was *d* = 0.81 (Note that, due to regulations in Sweden, patients are *not* automatically excluded from OMT due to illicit side use of substances).

**TABLE 1:** Background characteristics of participants (*n* = 48).

	Mean ± *SD* (range) or *n*	%
Age (years)	40.7 *±* 11.3 (23–66)	
Sex, male	35	73
*Birth country*		
Sweden	41	85
Other	7	15
*Residence*		
Private lease	20	42
Parent(s)’ apartment	10	21
Social services	9	19
Support apartment	5	10
Unhoused	2	4
Other	2	4
*Education*		
Elementary school	19	39
High school	22	46
University	7	15
*Employment*		
Full time (work)	5	10
Part time	3	6
Subsidised wage comp.	14	29
Pension	2	4
Unemployed	22	46
Institutionalised	2	4
*Social conditions*		
Married	3	6
Cohabiting	6	12
Single	39	81
Children	22	46

**TABLE 2:** Number and percentage of participants (*n* = 39) who have reached the criteria for each diagnosis according to MINI.

Psychiatric diagnosis (MINI)	*n*	%	Personality disorder (SCID‐II)	*n*	%
Depression	33	85	Borderline	33	85
ADHD	12	31	Antisocial	31	79
Panic disorder	12	31	Narcissistic	24	62
PTSD	11	28	Paranoid	22	56
Social anxiety	8	21	Compulsive	22	56
Agoraphobia	8	21	Depressive	21	54
OCD	7	18	Passive‐aggressive	21	54
Generalised anxiety disorder	6	15	Schizotype	17	44
Manic episode	5	13	Phobic	17	44
Psychotic symptoms	4	10	Independent	14	36
Hypomanic episode	1	3	Schizoid	10	26
Binge‐eating disorder	0	0	Histrionic	4	10
Bulimia	0	0			
Anorexia nervosa	0	0			

*Note*: Number and percentage of participants (*n* = 39) who reached the criteria for each personality disorder according to SCID‐II.

**TABLE 3:** Presence of substances in blood tests at admission (*n* = 41) and at the one‐year follow‐up (*n* = 41) for all participants for whom we have data, as well as the lower part of the table, showing all participants who completed the MAS interview (*n* = 15) at admission and follow‐up.

	*n* (%) Mean (*SD*)	*n* (%) Mean (*SD*)
	*Admission n = 41*	*One‐year follow‐up n = 41*
Opioids	41 (100%)	6 (15%)
Benzodiazepines	22 (54%)	20 (49%)
Stimulants	11 (27%)	8 (20%)
Cannabis	3 (7%)	4 (10%)
Empathogens	2 (5%)	0 (0%)
Alcohol (*n* = 26)	8 (35%)	5 (22%)
Total number of present substances	4.1 (2.3)	1.9 (1.9)***
	*Admission n = 15*	*One‐year follow‐up n = 15*
Opioids	15 (100%)	0 (0%)
Benzodiazepines	7 (27%)	5 (33%)
Stimulants	3 (20%)	2 (13%)
Cannabis	1 (7%)	0 (0%)
Empathogens	0 (0%)	0 (0%)
Alcohol (*n* = 26)	2 (13%)	0 (0%)
Total number of present substances	3.87 (1.92)	1.33 (1.76)**

*** *p* < .001. The effect size (*d*_*z*_) was 0.81;

** *p* = .002. The effect size (*d*_*z*_) was 1.01.

### Measures

Different dimensions of metacognition were measured through the semi‐structured interview, the Indiana Psychiatric Illness Interview (IPII) (Lysaker et al., 2002) and then analysed by the instrument MAS‐A. The sub‐scale *self‐reflectivity* refers to the capacity to examine one's own thoughts and emotions and to recognise them as shaped by both internal and external influences. *Understanding of others' minds* concerns the ability to consider and interpret the thoughts and feelings of other people. *Decentration* reflects the ability to view the world as a broader social context shaped by diverse perspectives and motives. Finally, *mastery* represents the capacity to apply self‐knowledge to manage and respond to psychological difficulties. The IPII interviews were conducted by the first author and two clinical psychology students trained by the first author. The IPII interview usually lasts between 30 and 60 minutes and is recorded. The interview includes questions such as “Tell us about your life up to now” and “Do you think you suffer from a mental health and/or drug or alcohol problem, and if so, what do you think it is?” For the participant's narrative to represent inherent metacognition, that is, what he or she can achieve in terms of reflection on life course and problems, the interviewer should listen actively but not introduce other material apart from what the participant brings up.

The transcript of the IPII is analysed and assessed using the MAS‐A, which assesses four different aspects of metacognition. *Self‐reflectivity* is assessed on a 10‐point scale (range 0–9) and measures the ability to reflect on one's own inner state. *Understanding of Others' Minds* is assessed on an 8‐point scale (range 0–7) and measures the ability to reflect on the inner states of others. *Decentration* is assessed on a 4‐point scale (range 0–3) and measures the ability to situate oneself and others within a broader social context with diverse perspectives and motives. *Mastery* is assessed on a 10‐point scale (range 0–9) and measures the ability to implement effective strategies for managing psychological problems and related disorders. The four subscales are combined to form a single overall scale (range 0–28) (Lysaker, Dimaggio, Carcione, et al., 2010; Wasmuth et al., 2015).

Thus, each scale consists of a series of levels that progress in order of complexity. For each scale, the rater assigns a score of 1 to each level that is fully achieved. A score of 0.5 can be assigned when levels are partially achieved. This process continues until no more levels can be attained (Semerari et al., 2003; Wasmuth et al., 2015). The MAS‐A has shown evidence of reliability, test–retest stability and validity (Hamm et al., 2012; Lysaker et al., 2005, 2010). To ensure the quality of the ratings, the first author and third author (another clinical psychologist specialising in addiction and therapy), both trained in the rating scale, independently conducted several parallel MAS‐A ratings of the transcripts. Subsequent comparison and deliberation of ratings ensured that the MAS‐A was applied accurately and consistently to the transcripts in the current study.

Executive function was assessed through a neuropsychological test battery using three tests from the executive function test battery D‐KEFS (Delis et al., 2001).

The D‐KEFS *verbal fluency test* (VFT) measures the ability of verbal fluency switching and production. The test provides a measure of the participant's ability to switch between different categories, speed in semantic retrieval and flow of verbal production. The participant's task is to verbally produce as many words as possible while alternating between two different categories for one minute. Outcome is measured in the number of correct switches between categories (switching) and correct words (production).

The D‐KEFS *trail making test B* (TMT) measures the ability of cognitive flexibility based on four basic functions: visual scanning, number sequencing, letter sequencing and motor speed. The participant's task is to draw a line as quickly as possible between numbers and letters in alternating order. Outcome is measured in completion time.

The D‐KEFS *twenty questions* measure the ability of problem solving. The test examines whether there are difficulties with basic cognitive functions such as perception, object recognition, visual discrimination and inhibition. It also shows impairments in the ability to assimilate oral feedback and problem‐solving. The test participant's task is to ascertain through as few questions as possible which figure the test leader chose from an image gallery. Outcome is measured in the total number of questions. Outcome raw scores for all tests are converted to scale points, where ≤7 points indicates cognitive impairment. Results are reported for measured executive functions, that is, verbal fluency, cognitive flexibility and problem‐solving.

BRIEF‐A (Behaviour Rating Inventory of Executive Function, Adult), a 75‐item questionnaire, was used to measure *self‐assessed executive function* (Roth et al., 2005). It contains statements such as “I make careless mistakes when performing tasks” and “I'm messy.” Results are presented as raw scores of the global executive function index, where higher scores indicate more problems.

The Structured Clinical Interview for DSM‐IV‐R Personality Disorder (SCID II) self‐report form (SCID‐II screen) was used to assess symptoms and types of personality disorders (First et al., 1997). The SCID‐II has displayed internal consistency coefficients ranging from acceptable to very good (0.35–0.83) and exhibits reasonable reliability (0.37–0.76) in substance use patients (First et al., 1997). The strong correlation between the SCID‐II interview and questionnaire supports using the questionnaire to reduce fatigue and non‐participation (Arntz, 1999).

MINI is a structured diagnostic interview used to inventory psychiatric diagnoses, including the diagnosis of addiction in accordance with DSM‐5 and ICD‐10 (Sheehan et al., 1998). Results are reported as the prevalence of diagnoses.

An excerpt of questions from the Addiction Severity Index (McLellan et al., 1980) was used to obtain sociodemographic information, including age at onset of drugs.

Current drug use was monitored using liquid chromatography–high resolution mass spectrometry (LC‐HRMS) analysis of oral fluid and alcohol marker B‐Peth for analysis of blood samples (Bergstrom et al., 2022).

### Procedure

Data collection took place during the initial phase of treatment at a day care clinic in western Sweden. Patients were approached and informed about the study, and written consent was obtained during the first week in the day care unit. An exclusion criterion for participation was the lack of ability to understand and speak Swedish, because participation required the ability to conduct interviews, cognitive tests and self‐assessment forms in Swedish. Exclusion criteria also ruled out patients considered too ill to participate—for example, those in acute phases of substance use or psychiatric conditions, as these states compromise a patient's ability to comprehend, consent to or adhere to the study protocol. Participants were recruited and either completed the first and second metacognition, treatment and drug tests assessments or declined to participate or did not attend the follow‐up session (See Flowchart of Participant recruitment flowchart in Figure 1). Neurological or learning disabilities were not screened for and were therefore not exclusion criteria for participation.

**FIGURE 1:**
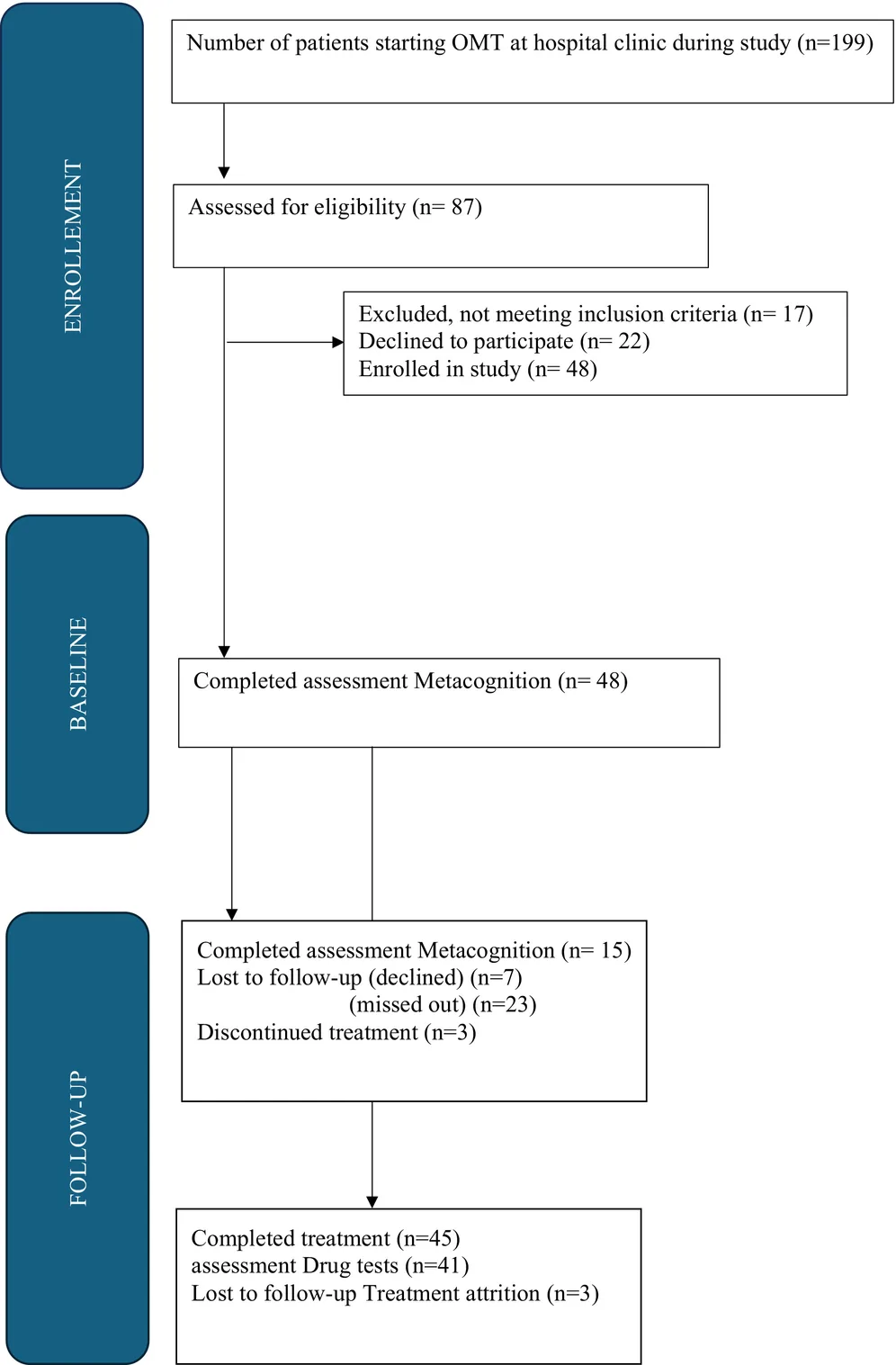
Flowchart of participant recruitment.

The study's data collection was a part of a larger data collection carried out on two separate occasions for the sake of the participants, as the procedure was cognitively demanding. The first occasion lasted between 60 and 120 min. It included the IPII, which was recorded and later transcribed and assessed using the MAS‐A, predating the cognitive testing, including three subtests from D‐KEFS. MAS‐A ratings were performed by the first author, who is trained in the use of the rating scale. The participants then filled out the BRIEF‐A and SCID‐II screening forms. The second occasion lasted between 30 and 60 min and included the structured interview MINI. IPII and MAS‐A were repeated at one‐year follow‐up.

Demographic information was obtained at the first and second occasions, and drug tests were routinely obtained and administered at treatment start by the staff; results were obtained by a researcher from the patient record.

The study protocol was approved by the Swedish Ethical Review Authority, DNR: 2020‐03753.

### Data processing and statistical analyses

SPSS version 28.0 was used for statistical analyses. The variable duration of drug use was computed by subtracting the participant's age from the age at onset of drug use. Descriptive statistics were calculated to provide means, standard deviations, percentages and ranges of demographic data, along with executive function and psychiatric and substance use data. The results were analysed according to the MAS‐A. Pearson correlations were used to examine correlations between variables. An independent samples *t*‐test was used to compare participants who completed a follow‐up assessment of metacognition with those who did not. A paired samples *t*‐test was used to examine longitudinal changes in metacognition among participants who completed the follow‐up assessment. We used Cohen's d for estimating the effect sizes. We also examined all variables using the corresponding non‐parametric tests (Wilcoxon signed‐rank test and Spearman's correlation test) to assess potential differences. No differences were observed; therefore, the results from the parametric analyses are reported. A *p*‐value of .05 was used to determine statistical significance in the analysis.

## RESULTS

### Metacognition at admission and follow‐up

The participants exhibited means of *self‐reflectivity* 5.83 (*SD* = 1.43), *understanding of others' minds* 3.7 (*SD* = 1.22), *decentration* 1.06 (*SD* = 0.70) and *mastery* 3.45 (*SD* = 1.20), along with a total MAS‐A score of 14.04 (*SD* = 3.36). Results for the present sample are shown in Figure 2, along with results from Kukla and Lysaker (2020) for a group of schizophrenia patients and Popolo et al. (2017) for a control group.

**FIGURE 2:**
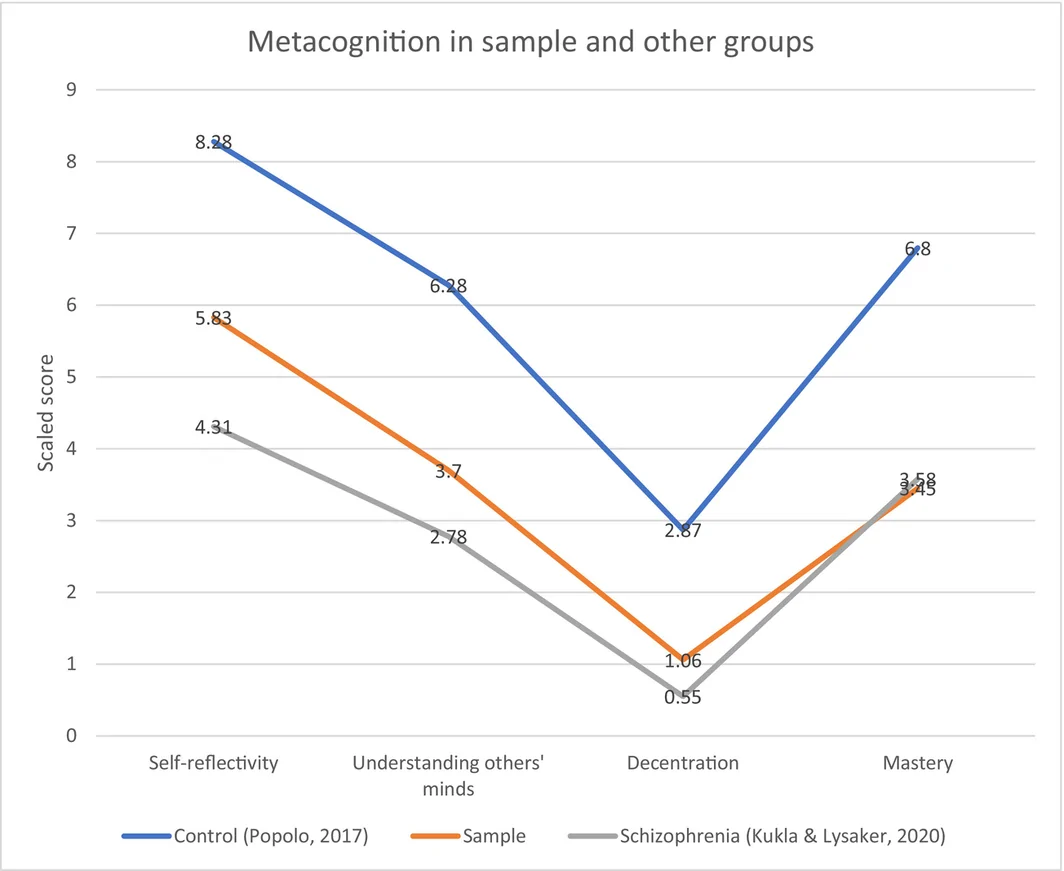
Sample baseline scaled score for MAS‐A subcategories *self‐reflectivity, understanding others' minds, decentration* and *mastery* (*n* = 48). Included for comparison are results from Kukla and Lysaker (2020) for a group of schizophrenia patients and Popolo et al. (2017) for a control group (*n* = 23).

Fifteen of the 48 participants completed the one‐year follow‐up MAS‐A interview. Comparisons between baseline data and follow‐up from the 15 participants who remained at the one‐year follow‐up are displayed in Table 4. There were no significant differences between admission and follow‐up regarding: *self‐reflectivity* (5.67 ± 1.33 vs. 5.90 ± 1.31; *p* = .550), *understanding of others' minds* (3.93 ± 1.28 vs. 3.47 ± 1.41; *p* = .084), *decentration* (1.13 ± .74 vs. .83 ± .79; *p* = .263), *mastery* (3.80 ± 1.28 vs. 3.43 ± .98; *p* = .354) and total MAS‐A score (14.53 ± 3.314 vs. 13.57 ± 3.023; *p* = .272). There were no differences in baseline metacognition between those who completed the follow‐up interview (*n* = 15) and those who did not (*n* = 33) (scores displayed in Table S1).

**TABLE 4:** Metacognition; subscales and total scores are compared between participants (*n* = 15) who have baseline values as well as one‐year follow‐up data.

	Baseline *n* = 15	One‐year follow‐up *n* = 15	Mean difference	95% CI	Effect size *d*_*z*_	*p*‐value (two‐sided)
Self‐reflectivity	5.67 (1.33)	5.90 (1.31)	−0.23	−1.05 to 0.58	−0.158	.550
Understanding others' minds	3.93 (1.27)	3.47 (1.40)	0.47	−0.07 to 1.01	0.480	.084
Decentration	1.13 (0.74)	0.83 (0.79)	0.30	−0.25 to 0.85	0.301	.263
Mastery	3.80 (1.28)	3.43 (0.98)	0.37	−0.45 to 1.19	0.247	.354
Total	14.53 (3.31)	13.57 (3.02)	0.97	−0.85 to 2.78	0.295	.272

*Note*: Mean differences, confidence intervals (95%), effect sizes and *p*‐values are presented.

### Correlations between metacognition and executive function

*Self‐reflectivity* exhibited a significant positive correlation with verbal fluency, *r*(46) = .321, *p* < .05. *Mastery* exhibited also a significant positive correlation with verbal fluency, *r*(46) = .352, *p* < .05, and *decentration* exhibited a significant negative correlation with self‐assessed executive function (BRIEF), *r*(40) = −.431, *p* < .01. There were no other significant correlations between metacognition and executive function (Table 5).

**TABLE 5:** Correlations of metacognition and subscales with executive functions (including self‐assessed executive function) (*n* = 46).

	Self‐reflectivity	Understanding others' minds	Decentration	Mastery	Total metacognition
*Executive function*					
Verbal fluency switching	.183	−.246	−.070	.218	.049
Verbal fluency production	.321*	−.101	.116	.352*	.252
Cognitive flexibility	.008	−.160	.109	.143	.016
Problem solving	.125	−.117	−.147	.111	.018
Self‐assessed executive function	−.042	.129	−.431**	.208	.007

* *p* < .05;

** *p* < .01.

## DISCUSSION

This exploratory pilot study aimed to investigate metacognition in patients with opioid use disorder (OUD) at treatment initiation and at a one‐year follow‐up, as well as to examine associations between metacognition and executive functioning at treatment onset. To our knowledge, this is the first study to assess metacognition and its longitudinal course in a sample of patients with OUD.

The sample of OMT patients with psychiatric comorbidity and polysubstance use showed varied metacognitive performance, as reflected by the mean baseline levels of the MAS‐A scales. Performance on the MAS‐A scale is rated and awarded points according to the level of complexity attained. According to the MAS‐A, the mean performance of the sample regarding *self‐reflectivity* and *understanding of others' minds* was rated below the scale level of understanding how thoughts and feelings impact behaviour in oneself and others, including intentions. Regarding *decentration*, the sample's performance was rated below the level required to understand the value of others' perspectives. Regarding *mastery*, the sample's performance was rated above the level of identifying a plausible problem; however, it was below the level of using behavioural or mental strategies to solve problems. (Lysaker, Dimaggio, Carcione, et al., 2010; Semerari et al., 2003; Wasmuth et al., 2015). The lack of normative values of the MAS‐A, however, limits further interpretation.

Previous research on metacognition has shown a distinct and generally low performance among schizophrenia patients and a generally higher level in control groups (Popolo et al., 2017). The present study group with opioid dependence showed a general level of metacognition that fell between that of the schizophrenia group and the control group, except for *mastery*, where the present group aligned with the schizophrenia group. The results of the OMT patient group in the present study are somewhat consistent with findings from previously studied groups with substance use disorders, in which similar patterns of metacognitive abilities have been observed (Wasmuth et al., 2015). Wasmuth et al.'s (2015) group of mainly polysubstance and alcohol dependence displayed slightly higher mean scale scores, but the same pattern of relatively pronounced *mastery* deficits. The group studied by Wasmuth et al. (2015) comprised mainly individuals with polysubstance and alcohol dependence (only two had opioid dependence) and was somewhat older than the current study group. The study did not report whether participants were abstinent at the time of the interview or the duration of their substance use. Together, these findings could indicate a general metacognitive profile among people with substance use disorders, which could be the subject of further research.

In the study by Lysaker et al. (2014), which examined the *mastery* subscale, the results also indicated higher mean scale scores than those observed in the present study. In Lysaker's study, the sample of mainly individuals with polysubstance and alcohol dependence (only two with opioid dependence) had the same mean age but about four years less substance use. Unlike the present sample, all participants were abstinent for at least one month, and no data on psychiatric comorbidities or personality disorders were provided.

In this pilot study, the metacognitive function in the OMT sample may be influenced by several factors that cannot be conclusively examined. The group had, on average, used substances for a prolonged period, and the majority had, despite opioid addiction, polysubstance use, which is the common pattern in OUD patients (Öhlin et al., 2011). Drugs generally impair brain function, which may partly explain the observed performance; however, distinguishing between the effects of different substances and psychiatric comorbidities is often difficult, as is typically the case in clinical settings (Ramey & Regier, 2019). Psychiatric comorbidity, including the prevalence of personality disorders, which was very common in this patient group, each associated with metacognitive impairment, may partially explain the results. Other potential explanations include poor nutrition, somatic illnesses and other psychiatric and neuropsychiatric disorders, which are also related to impairments (Strang et al., 2020). More research is needed in OUD and other SUD groups, with careful assessment of psychiatric comorbidities and participants' substance use status. Comorbidities and personality disorders may also affect metacognition and should be examined further.

The present findings suggest that metacognitive functioning remained largely stable over the one‐year follow‐up period. However, as this was a pilot study with a very small sample size, resulting in limited statistical power and potential selection bias, no clear conclusions can be drawn from the results. Ongoing substance use, including benzodiazepines, and treatment with opioids such as buprenorphine and methadone may have influenced the results. The role of psychiatric comorbidity or personality disorders also remains uncertain. Consequently, the observed trends should be interpreted cautiously. Non‐significant trends included an observed decrease in total metacognition. The subfunctions showed small declines, with understanding others' minds showing the most notable drop. Self‐reflectivity, however, increased slightly. These trends could be investigated further in larger samples.

Many participants still used substances, including benzodiazepines, which may suggest that these substances affected their metacognitive abilities and hindered improvement. The majority were treated with buprenorphine, and some with methadone, both of which are opioids that suppress brain function and could potentially contribute to the lack of improvement in metacognitive abilities. Although the patients had halved the number of different substances they used, this improvement may not have been reflected in enhanced metacognition. We also cannot determine whether psychiatric comorbidity and personality disorders contributed to the lack of significant change. The results from our previous study on executive functions in the same patient group (Högberg et al., 2025) showed that more complex functions, such as problem‐solving ability and cognitive flexibility, did not improve over time. One conclusion from this pilot study is that more comprehensive and systematic studies of metacognition, including other cognitive functions, are needed within OMT treatment.

Regarding the relation between metacognition and executive function, the present findings indicate a limited overall association between the two. The results showed positive associations between *self‐reflectivity* and *mastery* and executive functioning in verbal fluency production. One possible explanation is that the production aspect of verbal fluency reflects the ability to make statements about oneself. Thus, participants who generate more material about themselves will be rewarded in terms of metacognition regarding oneself (i.e., *self‐reflectivity* and *mastery*), but less so in terms of metacognition regarding others (i.e., *understanding others' minds* and *decentration*). Previous research on patients with schizophrenia shows that verbal fluency switching has been correlated with *mastery* (Lysaker et al., 2008). The moderate negative correlation between self‐assessed executive dysfunction and *decentration* may suggest that individuals reporting greater executive difficulties also show reduced capacity to adopt perspectives beyond their own mental states. Lysaker et al.'s (2008) study displayed low *decentration* being linked to worse performance of executive function compared to high *decentration* in a schizophrenia sample. The most pronounced relationship between metacognition and executive function in Lysaker et al.'s (2008) schizophrenia sample was problem solving, which displayed no correlation in the present sample. This may suggest a separate relationship between the functions in the two different samples with different pathologies, which requires further investigation, particularly with regard to the relationship between *decentration* and executive function in substance use.

In this patient group of individuals with OUD, there was a high prevalence of psychiatric comorbidity, both in terms of psychiatric diagnoses and the presence of personality disorders, consistent with findings from previous studies in this population (Öhlin et al., 2015; Strang et al., 2020). Common psychiatric diagnoses included depression, ADHD, panic disorder and PTSD. It was also common for participants to have more than one psychiatric diagnosis. Regarding personality disorders, the most frequently indicated types were borderline, antisocial and narcissistic personality disorders, which align with earlier findings in similar patient groups (Öhlin et al., 2015). It is imperative to regard comorbidity as symptoms that may both be parallel and affect one another, which need to be addressed in treatment (Popolo et al., 2025). Patients themselves often refer to substance use as a means to cope with agony and emotions, along with withdrawal (Grønnestad & Sagvaag, 2016). As mentioned in the background, the prevalence of comorbidities is one of the reasons why the concept of metacognition has been proposed for use in the research and clinical treatment of SUD. (Dimaggio et al., 2015). Previous research has shown an important link between pathology and deficits in patients with addiction, suggesting that a low level of *mastery* is associated with high levels of alexithymia and PD Cluster C traits in a substance use disorder (SUD) group (Lysaker et al., 2014). Both Wasmuth and the present study displayed similarly low levels of *mastery*, which may indicate that the metacognitive sub‐function is an important area for further study.

### Limitations

The limited sample size in the current study, particularly at follow‐up, is problematic, as it makes it difficult to generalise the findings to the broader patient population. In a larger sample, it would have been possible to conduct more detailed analyses of how factors constitute additional challenges to metacognitive functioning among individuals with OUD. On the other hand, this pilot study is the first study of its kind, serving as a foundation for more extensive future research in this area. Such research is also needed to explore the relationship between treatment outcomes and metacognitive abilities, an analysis that was not feasible in the present study due to the low number of participants. The frequency of comorbid problems, especially personality disorders and polysubstance use, along with the notably low level of executive functioning, might indicate an issue with the measure's discriminative ability. Refining the method is complicated by the group's heterogeneity and small sample size, which hinder subgroup analysis. The study's inclusive naturalistic scope provides challenges as diverse ages and frequent comorbid pathologies, polysubstance use or personality disorders, mirror limitations noted in previous research and the possibility to distinguish between comorbid pathologies. This also suggests that other functional limitations, such as learning disabilities, may be present but unnoticed and do not exclude participation. Using the IPII, which is narrative‐based, and the MAS‐A, a proven method in schizophrenia research, may mask the true level of functioning of the sample. However, research using other methods has found similar results in the SUD group (Gandolphe et al., 2018). Future research with larger samples could investigate differences between subgroups, as previous research on both psychosis and personality disorders shows distinct patterns of metacognitive impairment that warrant examination in the OMT context (Dimaggio et al., 2015; Lysaker et al., 2014). Unfortunately, this pilot study did not have access to information regarding the additional support patients may have received beyond their opioid maintenance treatment (OMT). Information about concurrent treatments, such as the type and duration of psychotherapy, would have provided valuable additional insight.

### Clinical importance

Treatment personnel in OMT should take into account that patients may have limited metacognitive abilities. It also appears that certain aspects, especially self‐assessed executive functioning, co‐vary with *decentration*, which may further contribute to difficulties for the patient. The clinical profile of many of these patients, therefore, may indicate limitations in integrating knowledge about the impact of thoughts and feelings on behaviour, understanding the intentions and agendas of others and utilising adequate coping strategies. These limitations may complicate the patient's ability to understand, formulate and carry out treatment plans, as well as to collaborate effectively with treatment personnel. Accurately assessing a patient's metacognitive capacities and executive functioning may enable clinicians to tailor interventions to the patient's actual skill level, thereby avoiding unrealistic demands and promoting achievable therapeutic progress (Dimaggio & Lysaker, 2020).

### Conclusion

The global opioid epidemic is deteriorating, and attrition from treatment is calling for ways to improve retention to save lives. Research displays considerable cognitive impairment, heavy psychiatric comorbidity and polysubstance use in OUD patients. To attribute the appropriate level of care, interventions need to be adapted to the patient's level of function. In this exploratory pilot study, metacognition provided a new perspective that adds to the understanding of OUD patients and highlights the relevance of metacognition as a potential target for future research and clinical interventions in OUD populations. In our sample of OMT patients, metacognitive performance was characterised by low levels of *mastery*, limited association with executive function and no improvement observed over the one‐year follow‐up period. However, as an exploratory study, these findings should be interpreted with caution.

## AUTHOR CONTRIBUTIONS

**John Hanson Högberg:** Conceptualization; investigation; writing – original draft; methodology; writing – review and editing; project administration. **Björn Philips:** Supervision. **Jonas Stålheim:** Methodology. **Kristina Berglund:** Methodology; writing – review and editing; supervision.

## CONFLICT OF INTEREST

The author(s) declared no potential conflicts of interest with respect to the research, authorship, and/or publication of this article.

## Supporting information


**Table S1.** Comparisons on baseline data between participants who were followed up (*n* = 15) and those who were not followed up regarding metacognition (*n* = 33). Means, standard deviations, mean differences, confidence intervals (95%), effect sizes and *p*‐values are presented.

## Data Availability

The data that support the findings of this study are available on request from the corresponding author. The data are not publicly available due to privacy or ethical restrictions.
