# Supplementary material for: Exploring metacognitive function in patients with opioid use disorder: A pilot study
Source: Psychol Psychother. 2026 Jun 1;99(4):1400–15. doi: 10.1111/papt.70081 (PMC13749256; doi:10.1111/papt.70081)
Supplement: Supplementary file 1 [file PAPT-99-1400-s001.docx]

Supplementary table 1. Comparisons on baseline data between participants who were followed up (n = 15) and those who were not followed up regarding metacognition (n = 33). Means, standard deviations, mean differences, confidence intervals (95 %), effect sizes and p-values are presented.

|  | Baseline data followed up  *n* = 15 | Baseline data not followed up  *n* = 33 | Mean difference | 95 % CI | Effect size Cohens d | P-value |
| --- | --- | --- | --- | --- | --- | --- |
| Self-Reflectivity | 5.67 (1.33) | 5.91 (1.49) | 0.24 | -.66 - 1.15 | .168 | .593 |
| Understanding Others’ Minds | 3.93 (1.27) | 3.59 (1.20) | -0.34 | -1.11 - 0.43 | -.279 | .374 |
| Decentration | 1.13 (0.74) | 1.03 (0.68) | -0.10 | -0.54 - 0.34 | -.147 | .640 |
| Mastery | 3.80 (1.28) | 3.29 (1.14) | -0.51 | -1.25 - 0.23 | -.433 | .171 |
| Total | 14.53 (3.31) | 13.82 (3.40) | -0.71 | -2.83 -1.40 | -.212 | .500 |
